# Are Twenty-Four Sessions of Aerobic Exercise Sufficient for Improving Cardiac Parameters in Diabetes Mellitus? A Randomized Controlled Trial 

**Published:** 2018-04

**Authors:** Soulmaz Rahbar, Sedigheh Sadat Naimi, Asghar Reza Soltani, Abbas Rahimi, Alireza Akbarzadeh Baghban, Nasrin khorami

**Affiliations:** 1 *School of Rehabilitation, Shahid Beheshti University of Medical Sciences, Tehran, Iran* *.*; 2 *Physiotherapy Research Center, School of Rehabilitation, Shahid Beheshti University of Medical Sciences, Tehran, Iran* *.*; 3 *Proteomics Research Center, School of Rehabilitation, Shahid Beheshti University of Medical Sciences, Tehran, Iran* *.*; 4 *Center of Diabetes, Imam Hospital, Hamedan University of Medical Sciences, Hamedan, Iran.*

**Keywords:** *Exercise*, *Diabetes mellitus*, *Physical fitness*

## Abstract

**Background:** Diabetes is a chronic disease that reduces cardiorespiratory fitness and increases systolic and diastolic blood pressures as well as resting heart rate due to the activity level of the sympathetic nervous system. The aim of this study was to assess the effectiveness of 2 types of aerobic exercise, with and without external loading, on cardiac parameters in diabetic patients.

**Methods**: This randomized controlled trial was carried out on 45 volunteers. These individuals were randomly divided into aerobic, weighted vest, and control groups. The aerobic protocol comprised 24 sessions of aerobic exercise. The exercise program for the weighted vest group was identical to that of the aerobic group, except that the subjects wore a weighted vest. The parameters were measured before and after the 24 sessions.

**Results**: The mean age of the study population was 48.30±5.02 years in the aerobic group, 48.33±5.74 years in the weighted vest group, and 48.60±4.79 years in the control group. Males comprised 7 (53.8%) patients in the aerobic group, 7 (58.3%) in the weighted vest group, and 8 (53.3%) in the control group. After 8 weeks, maximum oxygen consumption in the aerobic group (mean±SD=37.54±8.02 mL/kg/min, 95% CI: 5.48 to 11.60; P<0.001) and the weighted vest group (mean±SD=35.92±3.96 mL/kg/min, 95% CI: 4.36 to 9.64; P<0.001) was increased, similar to metabolic equivalent of task in the aerobic group (mean±SD=11.60±1.62 kcal/kg×h, 95% CI: 1.48 to 2.72; P<0.001) and the weighted vest group (mean±SD=11.21±1.11 kcal/kg×h, 95% CI: 1.23 to 2.28; P<0.001). Furthermore, resting heart rate decreased significantly in the aerobic group (mean ± SD=90.23±8.90 bpm, 95% CI: -13.93 to -1.29; P=0.022) and the weighted vest group (mean±SD=90.58±9.19 bpm, 95% CI: -0.16 to - 12.33; P=0.045).

**Conclusion**: These findings suggest that 24 aerobic exercise sessions might improve cardiac parameters in type 2 diabetes.

## Introduction

Diabetes is a chronic disease spread the world over.^        1 ^ Nearly, 1.6 million people are added to the diabetic patient population yearly^   2 ^ and one-third of diabetics are not recognized worldwide.^   3 ^ The prevalence of type 2 diabetes in Asia, Middle East, and Iran ranges from 1.2% to 14.6%, 4.6% to 40%, and 1.3% to 14.5%, respectively. Urbanization and lifestyle changes have contributed to the higher incidence of diabetes recently.^   4 ^ It has been stated that about 70% of diabetic mortalities are caused by coronary disease or stroke.^   3 ^

Higher blood pressure, higher resting heart rate, and lower maximum heart rate are among the symptoms seen in diabetic patients.^        5 ^ Moreover, diabetics are prone to nephropathy, retinopathy, neuropathy, and cardiovascular problems such as low systolic and diastolic blood pressures.^   6 ^ Exercise capacity is more important in determining mortality than other risk factors such as hypertension, dyslipidemia, obesity, gender, and smoking.^   7 ^ Exercise capacity is low in diabetics due to increased fat content, type 2 fibers, and oxidative glycolytic enzymes. In addition, impaired synthesis of glycogen, enzymatic changes to anaerobic formation, reduction in capillary density,^   8 ^ abnormal autonomic function with reduced cardiac output,^   9 ^ and effects of insulin resistance on mitochondrial function^   10 ^ might also be influential. 

Different methods have been proposed for assessing exercise capacity, among which metabolic equivalent of task (MET) and maximum oxygen consumption (VO_2_ max) are used more commonly. MET is a useful index for predicting exercise capacity.^   11 ^ It has been shown that reduced exercise capacity relative to healthy people is 20% in diabetic patients.^        1 ^ It has also been reported that patients have lower VO_2_ max than both similar nondiabetic men and women.^   12 ^ Additionally, cardiorespiratory fitness indicator has been considered a strong independent variable in mortality related to diabetes.^   8 ^

Obesity is another influential factor on type 2 diabetes. Central obesity has a greater role than general obesity in those patients; therefore, some anthropometric parameters such as hip circumference, waist circumference, waist-to-hip ratio, and waist-to-height ratio are more efficient markers than the body mass index (BMI) in predicting metabolic abnormalities.^        13 ^ According to the International Diabetes Federation (IDF), central obesity is an entrance criterion in metabolic syndrome.^        14 ^ Because of their shorter height and visceral obesity, the risk of metabolic disease is high in Asians (even with a normal BMI).^   15^

 Physical activity is the cornerstone in the treatment and prevention of diabetes. Inactivity increases the risk of death from heart problems.^   16 ^ Exercise decreases sympathetic activity and blood pressure by increasing glucose metabolism and insulin sensitivity.^   6 ^ Regardless of the beneficial effects of physical activity on hypertension, hypoglycemia, and dyslipidemia in patients with type 2 diabetes, physical activity is the only intervention that directly leads to an increase in cardiorespiratory fitness.^   8 ^ Aerobic exercise increases skeletal muscle capillary density and maximum oxygen consumption.^   17 ^

Most randomized control trials aimed at improving cardiac parameters have included patients with diabetes mellitus and cardiovascular disorders, with less emphasis being placed on the effects of aerobic training in type 2 diabetes. In addition, many studies have proposed long durations of exercise such as 12 weeks (Hatunic et al.,^   18 ^ Brandenburg et al.,^   19 ^ and Larose et al.^   20 ^), 6 months (Nishitani et al.^   21 ^), 9 months (Sénéchal et al.^   22 ^), and 12 months (Vanninen et al.^   23 ^). 

Undoubtedly, exercise in type 2 diabetes increases maximum oxygen consumption.^   8 ^ Despite several documents confirming the effectiveness of exercise, many diabetic patients do not tend to exercise. One possible reason is that exercise duration is too long to be done. Thus, assuming the impact of shorter exercise duration on cardiorespiratory fitness, in the present study, we considered the effects of 24 sessions of 2 types of aerobic exercise, with and without external loading, on cardiac and anthropometric parameters in patients with type 2 diabetes mellitus. We hypothesized that exercise with the weighted vest would improve cardiac health and anthropometrics in subjects with type 2 diabetes mellitus more than a simple aerobic exercise program. 

## Methods

The present parallel randomized control trial recruited participants through advertisement. The study protocol was approved by the Human Ethics Committee at Shahid Beheshti University of Medical Sciences, Tehran, Iran. The study was performed in accordance with the Declaration of Helsinki. Totally, 624 diabetic patients volunteered to participate in this study. The study population was evaluated based on our inclusion criteria, comprised of 2 to 10 years’ history of having diabetes, age between 40 and 60 years (males or females), HbA1c value of 6% to 10%, BMI of 20 to 30 kg/m^2^, inactive lifestyle (<30 min per week of exercise), not smoking, not drinking alcohol, not using opium, not using insulin injections, no hypertension, and no history of cardiovascular, muscular, skeletal, neurological, or metabolic diseases. The exclusion criteria consisted of absence for 2 successive sessions, respiratory problems during exercise, and inability to follow the aerobic group’s protocol. The contemporaneous criterion for the control group (free from intervention) was regular exercise more than 30 minutes per week. The sample size was 12 individuals calculated according to a study by Maiorana et al.^        24^

**Figure 1:**
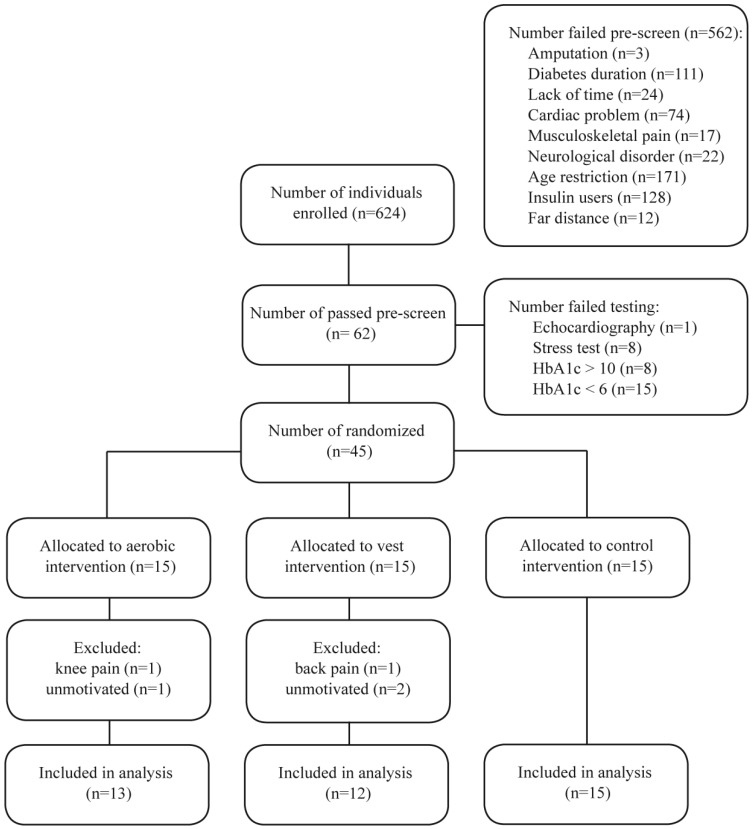
CONSORT diagram, showing the result of randomization and patient enrollment.

Forty-five volunteers met the inclusion criteria. All the participants filled out and signed the informed consent. Figure 1 depicts the various stages of this study in a CONSORT diagram. This trial study met the criteria in the CONSORT checklist.

The training program was done by the first author. The aerobic protocol comprised 24 sessions (8 wk) of aerobic exercise on a treadmill (MOTORIZED TREADMILL^®^, OMEGA GT, USA) with 0 slope, 3 days per week for 30 minutes. The intensity of the training protocol was 50% to 70% maximum heart rate. Maximum heart rate was estimated using the Bruce protocol test. 

The intensity of exercise was gradually improved during the 8 weeks. The exercise program for the weighted vest was identical to that of the aerobic group, except that the subjects wore a weighted vest before standing on the treadmill. The initial inner weight of the vest was 2% of the subject’s body weight and it was gradually increased to 5% of the subject’s body weight. 

To avoid any complications if the blood glucose level was lower than 100 mg/dL, we administered 15 g of carbohydrate or food supplements. Blood glucose was measured again after 20 to 30 minutes. Heart rate was monitored throughout the training program using a digital heart rate meter by placing the belt around the chest and adjusting the heart rate monitor like a wrist watch (Beurer^®^PM60 digital pulse meter, Germany). 

Cardiac parameters of MET, resting heart rate, maximum functional heart rate, and systolic and diastolic blood pressures were measured before and after the 24 sessions. The exercise test was started based on the standard Bruce protocol. The time was recorded when the patient became fatigued and could no longer continue the exercise. Total exercise time was calculated via a specific formula for the males and females. Each participant’s maximum oxygen consumption (VO_2_ max) was calculated via VO_2_ max=14.8–(1.379×T)+(0.451×T2)–(0.012×T3) for the men and VO2 max=4.38×T–3.9 for the women. Note that T is “total running time” in minutes.^   25 ^ The MET value at the various stages of the exercise test was recorded automatically moment by moment. To prevent overestimating MET, we recorded the highest value immediately after the subject reached volitional fatigue. 

Weight was recorded on a digital scale (Omron^®^HN289 digital personal scale, China), height was measured with a stadiometer (WB-800H,Tanita,USA), BMI was calculated as weight (kg)/height^2^ (m), waist circumference was measured at the midpoint between the lower margin of the last palpable rib and the top of the iliac crest with a flexible tape (Seca®210, China), hip circumference was measured around the widest portion of the buttocks with the tape parallel to the floor, and finally waist-to-height ratio and waist-to-hip ratio were calculated as waist/height and waist/hip, respectively.^        13^

The statistical software SPSS, version 16.0, was used for all the statistical analyses. The statistical analyses were performed based on the “per protocol” approach. To test normality, that is, to verify if the distribution of the data was parametric, we used the Kolmogorov–Smirnov test. The paired *t*-test was applied to determine the differences between the variables before and after the 8 weeks of intervention in each group. ANCOVA and the Bonferroni test were employed to compare the variables after the 8 weeks between the 3 groups. Additionally, ANCOVA was applied to estimate the effect size (partial eta squared).The significance level was 0.05.

## Results

**Figure 2:**
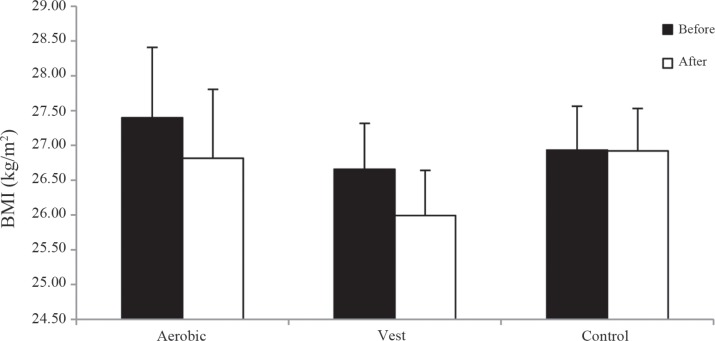
Body mass index (BMI) changes before and after intervention in the aerobic, weighted vest, and control groups

**Figure 3:**
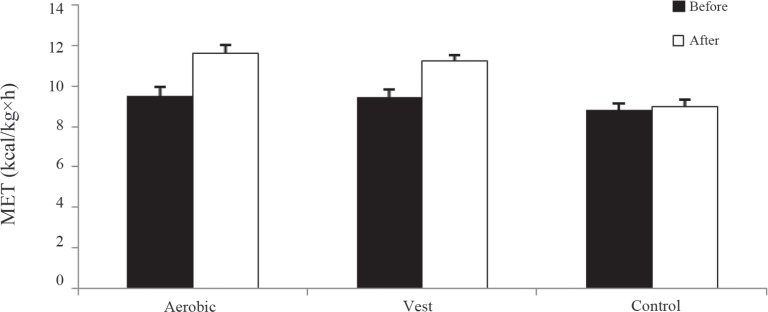
Metabolic equivalent of task (MET) changes before and after intervention in the aerobic, weighted vest, and control groups

The study was started with 45 patients with type 2 diabetes (insulin independent), but 40 patients (15 in the control group [8 males and 7 females], 13 in the aerobic group [7 males and 6 females], and 12 in the weighted vest group [7 males and 5 females]) completed the study. The mean±SD age was 48.31±5.02 years in the aerobic group, 48.33±5.74 years in the weighted vest group, and 48.60±4.80 years in the control group. The results exhibited no significant differences at baseline in the BMI (P=0.806), sex (P=0.965), age (P=0.986), duration of diabetes (P=0.555), maximum ejection fraction (P=0.227), and MET (P=0.382) (Tables 1-4). The trends of the BMI and MET changes are shown in Figure 2 and Figure 3. 

After 8 weeks of training, weight, hip circumference, waist circumference, BMI, and waist circumference/height were significantly different between the 2 training groups: aerobic with and without the weighted vest (Table 1). There was a significant difference in waist circumference / hip circumference in the weighted vest group by comparison with baseline.

After 8 weeks, VO_2_ max in the aerobic group (mean±SD=37.54±8.02* kcal/kg×h* 95%CI: 5.48 to 11.60; P<0.001) and the weighted vest group (mean±SD=35.92 ±3.96* kcal/kg×h*, 95%CI: 4.36 to 9.64; P<0.001) in addition to MET in the aerobic group (mean±SD=11.60±1.62, 95% CI: 1.48 to 2.72; P<0.001) and in the weighted vest group (mean±SD=11.21±1.11, 95% CI: 1.23 to 2.28; P<0.001) were significantly different between the 2 training groups and the control group. Further, the resting heart rate was significantly different between the aerobic group (mean±SD=90.23±8.90, 95% CI: -13.93 to -1.29; P=0.022) and the weighted vest group (mean±SD=90.58±9.19, 95% CI: -0.16 to -12.33; P=0.045). 

**Table 1:** Comparison of the mean difference in the anthropometric data between the 3 groups before and after 8 weeks*

	Aerobic Group				Weighted Vest Group				Control Group			
	Pre	Post	Difference (95% CI)	P	Pre	Post	Difference (95% CI)	P	Pre	Post	Difference (95% CI)	P
Weight (kg)	13.64±75.80	13.23±74.17	-1.64 (-2.40 to -0.88)	< 0.001	74.73±8.36	72.87±8.29	-1.85 (-2.74 to -0.97)	0.001	9.91±75.03	74.96±9.59	-0.07 (-0.52 to 0.39)	0.759
HC (cm)	105.08±7.71	6.16±101.15	-3.92 (-5.79 to -2.05)	0.001	105.50±8.18	100.58±6.80	-4.92 (-6.76 to -3.07)	<0.001	7.90±106.73	106.53±7.79	-0.20 (-0.86 to 0.47)	0.531
WC (cm)	10.81±95.08	10.36±90.38	-4.69 (-6.82 to -2.56)	< 0.001	95.33±7.56	88.75±8.11	-6.58 (-8.88 to -4.27)	<0.001	16.96±99.87	17.34±100.07	0.20 (-0.39 to 0.79)	0.486
BMI (kg/m^2^)	3.65±27.40	3.58 ±26.81	-0.58 (-0.85 to -0.31)	0.001	26.65±2.30	25.99±2.26	-0.66 (-0.99 to -0.33)	0.001	2.42±26.93	2.37±26.92	-0.01 (-0.17 to 0.14)	0.849
WC/HC (cm)	0.06±0.90	0.06±0.89	-0.01 (-0.02 to 0.003)	0.125	0.90±0.07	0.88±0.06	-0.02 (-0.05 to 0.01)	0.105	0.18±0.94	0.18±0.94	0.003 (-0.001 to 0.01)	0.173
WC/H (cm)	0.06±0.57	0.05±0.54	-0.03 (-0.04 to -0.01)	< 0.001	0.57±0.44	0.53±0.41	-0.04 (-0.05 to -0.02)	<0.001	0.09±0.60	0.10±0.60	0.001 (-0.002 to 0.01)	0.450

HC, Hip circumference; WC, Waist circumference; BMI, Body mass index; WC/HC, Waist circumference/hip circumference; WC/H, Waist circumference/height

* Data are presented as mean±SD.

**Table 2:** Comparison of the mean difference in the anthropometric data between the 3 groups after 8 weeks

	Aerobic vs. Weighted Vest		Aerobic vs. Control		Weighted Vest vs. Control		
	Mean Difference (95% CI)*	P**	Mean Difference (95% CI)*	P**	Mean Difference (95% CI)*	P**	Partial eta squared
Weight (kg)	0.25 (-0.87 to 1.38)	1.000	-1.55 (-2.61 to -0.49)	0.002	-1.80 (-2.88 to -0.71)	0.001	0.372
HC (cm)	0.93 (-1.26 to 3.11)	0.881	-3.99 (-6.06 to -1.91)	<0.001	-4.91 (-7.03 to -2.80)	<0.001	0.526
WC (cm)	1.89 (-1.06 to 4.83)	0.349	-4.95 (-7.78 to -2.12)	<0.001	-6.84 (-9.72 to -3.96)	<0.001	0.519
BMI (kg/m^2^)	0.11 (-0.31 to 0.53)	<0.999	-0.55 (-0.95 to -0.16)	0.004	-0.66 (-1.07 to -0.25)	0.001	0.360
WC/HC (cm)	0.01 (-0.02 to 0.04)	<0.999	-0.02 (-0.04 to 0.01)	0.501	-0.03 (-0.06 to 0.001)	0.068	0.139
WC/H (cm)	0.01 (-0.01 to 0.03)	0.362	-0.03 (-0.05 to -0.01)	<0.001	-0.04 (-0.06 to -0.02)	<0.001	0.515

HC, Hip circumference; WC, Waist circumference; BMI, Body mass index; WC/HC, Waist circumference/hip circumference; WC/H, Waist circumference/height

* Mean difference (95% CI): post intervention minus pre intervention (lower bound, upper bound)

** Resulted from analysis of covariance (ANCOVA)

**Table 3:** Comparison of the mean difference in the cardiac parameters between the 3 groups before and after 8 weeks*

	Aerobic Group				Weighted Vest Group				Control Group			
	Pre	Post	Difference (95% CI)	P	Pre	Post	Difference (95% CI)	P	Pre	Post	Difference (95% CI)	P
VO_2_ max (mL/kg/min)	7.67±29.00	8.02±37.54	8.54 (5.48 to 11.60)	<0.001	3.96±28.92	39.23±3.90	7.00 (4.36 to 9.64)	<0.001	3.44±26.60	5.57±27.93	1.33 (-1.27 to 3.94)	0.290
MET (kcal/kg/ hour )	9.50±1.68	11.60±1.62	2.10 (1.48 to 2.72)	<0.001	9.45±1.30	11.21±1.11	1.76 (1.23 to 2.28)	<0.001	8.84±1.17	8.97±1.28	0.13 (-0.47 to 0.74)	0.647
Max heart rate (bpm)	12.89±64.69	9.69±169.54	4.85 (-2.12 to 11.81)	0.155	12.45±158.75	164.25±15.15	5.50 (-1.38 to 12.39)	0.107	15.07±154.13	14.67±155.47	1.33 (-3.42 to 6.08)	0.557
Resting heart rate (bpm)	97.85±12.35	8.90±90.23	-7.61 (-13.93 to -1.29)	0.022	8.72±96.83	90.58±9.19	-6.25 (-0.16 to -12.33)	0.045	14.26±94.00	6.35±92.93	-1.06 (-7.31 to 5.18)	0.720
SBP (mmHg)	8.62±119.23	9.54±120.77	1.54 (-4.42 to 7.50)	0.584	7.38±120.00	120.83±10.84	0.83 (-4.88 to 6.55)	0.754	7.84±120.00	7.56±120.00	-0.71 (-6.90 to 5.47)	0.807
DBP (mmHg)	5.55±78.46	10.13±77.69	-0.77 (-6.53 to 4.99)	0.776	5.77±78.33	78.33±5.77	0.00 (-2.70 to 2.70)	<0.999	5.56±78.20	7.23±78.00	0.00 (-4.53 to 4.53)	<0.999

SBP, Systolic blood pressure; DBP, Diastolic blood pressure; MET, Metabolic equivalent of task

* Data are presented as mean±SD.

**Table 4:** Comparison of the mean difference in the cardiac parameters between the 3 groups after 8 weeks

	Aerobic vs. Weighted Vest		Aerobic vs. Control		Weighted Vest vs. Control		
	Mean Difference (95% CI)*	P**	Mean Difference (95% CI)*	P**	Mean Difference (95% CI)*	P**	Partial eta squared
VO_2_ max (mL/kg/min)	1.56 (-3.06 to 6.18)	>0.999	7.72 (3.26 to 12.18)	<0.001	6.16 (1.62 to 10.71)	<0.001	0.371
MET (kcal/kg/ hour )	0.36 (-0.59 to 1.30)	>0.999	2.15 (1.24 to 3.06)	<0.001	1.80 (0.87to 2.72)	<0.001	0.531
Max heart rate (bpm)	1.13 (-8.64 to 10.90)	>0.999	6.68 (-2.92 to 16.29)	0.268	5.55 (-3.85 to 14.96)	0.441	0.091
Resting heart rate (bpm)	-0.71 (-7.76 to 6.35)	>0.999	-4.05 (-10.78 to 2.69)	0.420	-3.34 (-10.19 to 3.51)	0.687	0.068
SBP (mmHg)	0.24 (-8.64 to 9.12)	>0.999	1.79 (-6.75 to 10.33)	<0.999	1.55 (-7.17 to 10.26)	<0.999	0.009
DBP (mmHg)	-0.70 (-7.86 to 6.45)	>0.999	-0.45 (-7.34 to 6.44)	<0.999	0.25 (-6.79 to 7.29)	<0.999	0.002

SBP, Systolic blood pressure; DBP, Diastolic blood pressure; MET, Metabolic equivalent of task

* Mean difference (95% CI): post intervention minus pre intervention (lower bound, upper bound)

** Resulted from analysis of covariance (ANCOVA)

Two patients were excluded because of acute knee pain and acute back spasm after 12 and 11 sessions, respectively. The reason for acute pain was walking on the treadmill during the intervention program. One patient fainted because of hypoglycemia and the program was stopped just for 1 session. No other harm effects happened during the course of this intervention.

## Discussion

In the present study, the effects of 24 sessions of aerobic exercise on cardiac and anthropometric parameters were assessed in diabetic patients. Our findings revealed positive effects of 8 weeks of aerobic training on resting heart rate, aerobic capacity, and anthropometric parameters compared to baseline in these 2 exercise groups.

Systolic and diastolic blood pressures are considered important cardiovascular parameters in diabetic patients. In our study, systolic and diastolic pressures in the 2 training groups were unchanged, which is consistent with a study by Fritz et al.^   26 ^ The reason for the absence of change in blood pressure in our study despite 24 sessions of aerobic exercise could be attributed to normal blood pressure values. The change percent of systolic and diastolic blood pressures was negligible. Nonetheless, in the study by Fritz et al.,^   26 ^ insufficient exercise intensity was the reason behind the unchanged blood pressure. Yan et al.^   6 ^ reported that 12 weeks of training with low-intensity (50% VO_2_ max) and high intensity (75% VO_2_ max) reduced blood pressure. We found no changes in blood pressure because the level of blood pressure in all our patients was within the normal range. Training type, exercise duration, and participants encompass some differences between these studies. 

Aerobic training reduces resting heart rate through increasing the activity of the parasympathetic nervous system and decreasing intrinsic heart rate.^   27 ^ Liu et al.^         28 ^ and Fahlman et al.^   29 ^ respectively reported that resting heart rates declined after 12 and 10 weeks of aerobic and resistance exercise in patients with type 2 diabetes and heart rate recovery problems. A study reported that 12 weeks of resistance training and yoga/stretching in overweight women did not change resting heart rate because a 3-month exercise duration was too short to cause a change and, therefore, long-term exercise is essential.^   30 ^ Conversely, Castaneda et al.^   31 ^ documented no difference in resting heart rate after training in their study population. This difference may, however, be attributed to resistance exercise which the authors implemented. Elsewhere, Braz et al.^   32 ^ reported that 3 weeks of walking with intensity of 55% to 65% of heart rate reserve and for 30 to 55 minutes did not change resting heart rate in patients with hypertension patients. Thus, it seems that more than 3 weeks is required to reduce resting heart rate. A previous study showed that 4 weeks of training changed resting heart rate in stage-1 hypertensive patients.^   33 ^ In another investigation, high-intensity interval training decreased resting heart rate after 12 weeks in women with insulin resistance.^   34 ^ Type of exercise, duration, intensity, and target groups may have resulted in these controversial results.

In the current study, after 24 sessions, there was a 7% decline in resting heart rate in the aerobic group, 6% in the weighted vest group, and 0.1% in the control group. Nevertheless, resting heart rate in the 2 aerobic groups was not significantly different from that in the control group.

 Different resting heart rate ranges have been known to correlate with different mortality risk factors. Resting heart rates between 51 and 80 bpm have been associated with a 40% to 50% risk increase, while resting heart rates between 81 and 90 bpm cause a twofold risk increase and this could rise to threefold when resting heart rates exceed 90 bpm relative to the lowest heart rate category (<50 bpm). In the current study, the patients had resting heart rates higher than 90 bpm; as a result, the decrease in resting heart rate in the aerobic and weighted vest groups was associated with reduced risk.^   35^

One of the most valuable criteria for the evaluation of cardiac function is maximum functional heart rate. A percentage of this parameter is used to prescribe exercise intensity. Obtaining maximum heart rate is not the aim in many studies. Age is of the greatest importance in that it influences maximum heart rate. Therefore, the 220-x formula (x=age), which is an estimation method, is used. Sex and exercise activity are reported to exert less effect on maximum heart rate.^   36 ^ The aim of our training protocol was not to fasten heart rate but to enhance the autonomic nervous system activity, with a view to augmenting maximum functional heart rate after 24 sessions of training. Cardiac adaptation to training confers better sympathetic and parasympathetic activity and faster return to normal cardiovascular adaptation.^   37 ^ Maximum functional heart rate is lower in diabetic patients than in healthy subjects.^   38 ^ Increasing maximum functional heart rate is followed by reduced morbidity.^   11 ^ There are different mechanisms involved. Synchronization of the autonomic nervous system (parasympathetic and sympathetic) increases resting heart rate to maximal exercise. Furthermore, reduced parasympathetic nervous activity (vagal withdrawal) enhances heart rate during exercise and then, sympathetic nervous activity starts to increase heart rate above 100 bpm.^   39 ^

In the present study, we calculated maximum functional heart rate based on the Bruce protocol. We also conducted stress test to assess physical capacity and carried out different field tests to evaluate functionality. After 24 sessions, the improvement rates in maximum functional heart rate in the aerobic group, weighted vest group, and control group were 0.03%, 0.03%, and 0.01%, respectively. Nevertheless, maximum functional heart rate in the 2 aerobic groups was not significantly higher than that in the control group. Kim et al.^11^ reported an increasing trend of maximum heart rate in diabetic and nondiabetic patients with myocardial infarction after 8 weeks of cardiac rehabilitation. Elsewhere, another investigation indicated that the increase of this parameter was obvious in long-standing type 2 diabetes after 12 weeks of resistance training.^   37 ^

Exercise improves aerobic capacity and reduces cardiovascular risk by increasing insulin sensitivity, enhancing glycemic control,^   40 ^ augmenting cardiac output, increasing adaptation enzymes and mitochondrial mass, increasing mitochondrial enzyme content changes,^     41 ^ and enhancing endothelial function.^   42 ^ It has been documented that MET has a strong positive relationship with cardiorespiratory fitness.^   22 ^ In the current study, increased MET and VO_2_ max rates of 23% and 19% in the aerobic group and 32% and 25% in the weighted vest group were the consequence of 24 exercise sessions. This is while the control group’s MET and VO_2_ max increased just by 2% and 5%, which could be attributed to the familiarity of those participants with the exercise test protocol. Given that low fitness is an efficient indicator of mortality from heart disease, a 1-unit rise in MET is associated with 13% mortality reduction.^   7 ^ Thus, it can be concluded that the increase of 2 METs in the aerobic and weighted vest groups may reduce their mortality rates. Increasing exercise capacity in our study is congruent with previous investigations.^17^^-^^22^ Intervention duration, to increase aerobic capacity, was different among other investigations ranging from 3 months,^17^^, ^^18^ 6 months,^20^ and 9 months^   22 ^ to 12 months.^   23 ^ Of course, it is probable that positive results would be obtained by a longer duration of exercise, but the aim of the current study was to show improvement in exercise capacity in shorter time periods with a view to obtaining positive results more quickly. Asa et al.^42^ and Schreuder et al.^   43 ^ utilized 8 weeks of training to increase aerobic capacity. However, the participants in the former study were trained under special situations of increase and decrease in oxygen, whereas the authors of the latter study utilized hydrotherapy. Determining the amount of oxygen is possible only at a laboratorial environment and using hydrotherapy is not possible for some individuals. We performed the present study using the treadmill so that the results could be extrapolated to fast walking. Fritz et al.^   26 ^ reported that 4 months of exercise exerted no effect on aerobic capacity. The fact that the authors failed to determine the intensity of walking may have been the reason behind the absence of any change in this parameter.

The good rate of changes in our study population’s anthropometric parameters is the consequence of the 8-week intervention. In the current study, the participants’ body weight, BMI, pelvic circumference, waist circumference, and waist / height ratio were reduced by 2.14%, 2.11%, 3.62%, 4.88%, and 4.88% in the aerobic group and 2.48%, 2.49%, 4.56%, 6.92%, and 6.92% in the weighted vest group after the 24 training sessions respectively compared to slight changes in the control group. Only waist circumference/hip circumference parameter change percent ratio was low (-1.29%, -2.42%, and 0.35% in the aerobic, weighted vest, and control groups, correspondingly). Contrary to our findings with respect to changes in anthropometric variables, Hatunic et al.^   18 ^ documented that waist-to-hip ratio, weight, and BMI were unchanged after 3 months of aerobic exercise among their study subjects. Likewise, Donovan et al.^   44 ^ reported no significant changes in weight and waist circumference after 24 sessions. It appears that individual differences in terms of height, weight, and BMI led to such differences. Contrarily, reduced waist circumference and no change in BMI were reported after cardiac rehabilitation by Nishitani et al.^   21 ^

The results of the current study are generalizable to inactive lifestyle males or females only with a 2- to 10-year history of type 2 diabetes (40–60 years old). This study does not guarantee improvement in cardiac parameters in any type of diabetes with cardiovascular, muscular, skeletal, neurological, and other disorders. Anticipating improvement in the cardiovascular system in diabetics with HbA1c values greater than 10% or less than 6% is questionable. 

One of the main limitations of the present study was fatigue in the study participants due to long periods of assessment (pre-evaluation endocrinology visits, cardiology visits, echocardiography, and exercise testing) and intervention (24 sessions). 

Many previous studies used simple aerobic or resistance exercises, but the novel aspect of the current study was that 1 group of aerobic exercise was specified with weighted vests.

We recommend that higher intensity exercises and fewer sessions be used in future studies. What such future investigations should also take into consideration is the measurement of the time of the stress test.

## Conclusion

The present study showed that 24 sessions of aerobic exercise with and without weighted vests caused changes in cardiac parameters among diabetic patients, while there was no change in the parameters among the control group or warning signs for sedentary patients. The weighted vest did not have any significant effect on cardiac and anthropometric parameters in the aerobic exercise group except waist circumference/hip circumference. Thus, higher intensive exercise regimes could be taken into account to maintain a healthy cardiovascular function and reduce complications and mortality.

## Notes:

***This paper should be cited as: ***Rahbar S, Naimi SS, Soltani AR, Rahimi A, Akbarzadeh Baghban A, Khorami N. Twenty-Four Sessions of Aerobic Exercise with and without External Loading Are Sufficient for Improving Cardiac Parameters in Subjects with Type 2 Diabetes Mellitus: A Randomized Controlled Trial. J Teh Univ Heart Ctr 2018;13(2):43-51.
